# Inhibition of hypothalamic leukemia inhibitory factor exacerbates diet-induced obesity phenotype

**DOI:** 10.1186/s12974-017-0956-9

**Published:** 2017-09-02

**Authors:** Milena Fioravante, Bruna Bombassaro, Albina F. Ramalho, Nathalia R. Dragano, Joseane Morari, Carina Solon, Natalia Tobar, Celso D. Ramos, Licio A. Velloso

**Affiliations:** 1 Laboratory of Cell Signaling, University of Campinas, Campinas, São Paulo 13084-970 Brazil; 2 Department of Radiology, University of Campinas, Campinas, São Paulo 13084-970 Brazil

**Keywords:** Diabetes, Glucose, Insulin, Adipose tissue, Inflammation

## Abstract

**Background:**

The consumption of large amounts of dietary fats can trigger an inflammatory response in the hypothalamus and contribute to the dysfunctional control of caloric intake and energy expenditure commonly present in obesity. The objective of this study was to identify chemokine-related transcripts that could be involved in the early stages of diet-induced hypothalamic inflammation.

**Methods:**

We used immunoblot, PCR array, real-time PCR, immunofluorescence staining, glucose and insulin tolerance tests, and determination of general metabolic parameters to evaluate markers of inflammation, body mass variation, and glucose tolerance in mice fed a high-fat diet.

**Results:**

Using a real-time PCR array, we identified leukemia inhibitory factor as a chemokine/cytokine undergoing a rapid increase in the hypothalamus of obesity-resistant and a rapid decrease in the hypothalamus of obesity-prone mice fed a high-fat diet for 1 day. We hypothesized that the increased hypothalamic expression of leukemia inhibitory factor could contribute to the protective phenotype of obesity-resistant mice. To test this hypothesis, we immunoneutralized hypothalamic leukemia inhibitory factor and evaluated inflammatory and metabolic parameters. The immunoneutralization of leukemia inhibitory factor in the hypothalamus of obesity-resistant mice resulted in increased body mass gain and increased adiposity. Body mass gain was mostly due to increased caloric intake and reduced spontaneous physical activity. This modification in the phenotype was accompanied by increased expression of inflammatory cytokines in the hypothalamus. In addition, the inhibition of hypothalamic leukemia inhibitory factor was accompanied by glucose intolerance and insulin resistance.

**Conclusion:**

Hypothalamic expression of leukemia inhibitory factor may protect mice from the development of diet-induced obesity; the inhibition of this protein in the hypothalamus transforms obesity-resistant into obesity-prone mice.

**Electronic supplementary material:**

The online version of this article (10.1186/s12974-017-0956-9) contains supplementary material, which is available to authorized users.

## Background

The consumption of large portions of dietary fats is one of the most important environmental factors leading to obesity [1]. Experimental studies have shown that, in addition to their caloric value, which could, per se, lead to a positive energy balance, dietary fats can also trigger hypothalamic inflammation and induce the damage to key neurons involved in the control of food intake and energy expenditure, further increasing anabolism [2–4]. An important event during the early stages of obesity associated hypothalamic inflammation is the recruitment of bone marrow-derived monocytes to compose the macrophage/microglial network that sustains chronic inflammation [5]. Neuronal-derived CX3CL1 (fractalkine) was the first chemokine shown to play a role in this process [5]; it is rapidly induced after the introduction of a high-fat diet (HFD), and its inhibition reduces the recruitment of bone marrow-derived monocytes and attenuates diet-induced hypothalamic inflammation [5].

As in other chronic inflammatory processes [6–8], it is expected that in the hypothalamus, a network of chemokines and cytokines orchestrate the installation and perpetuation of inflammation that occur in response to the consumption of dietary fats. Here, we hypothesized that different landscapes of chemokines produced in the hypothalamus in response to dietary fats could play a role in the protection against or predisposition to obesity. To test this hypothesis, we initially evaluated the landscape of chemokine-related transcripts expressed in the hypothalamus of mice fed on a HFD, comparing obesity-prone (OP) versus obesity-resistant (OR) mice [9]. We found that leukemia inhibitory factor (LIF) was one of the transcripts with the greatest difference between the two groups, with high expression in the hypothalamus of OR and low expression in the hypothalamus of OP mice.

LIF is an interleukin-6 class cytokine that modulates myeloid cell differentiation [10]. Studies have shown that LIF can act upon proopiomelanocortin (POMC) neurons to induce an anorexigenic response [11, 12]. With this information in mind, we next hypothesized that different levels of LIF expression in the hypothalamus of mice fed a HFD could affect the predisposition to obesity by regulating both neurotransmitter and inflammatory protein expression. To test this hypothesis, we inhibited hypothalamic LIF in mice fed a HFD and, by doing so, we transformed OR into OP mice. Thus, LIF expressed in the hypothalamus emerges as an early determinant of obesity predisposition.

## Methods

### Animals

**Fig. 1:**
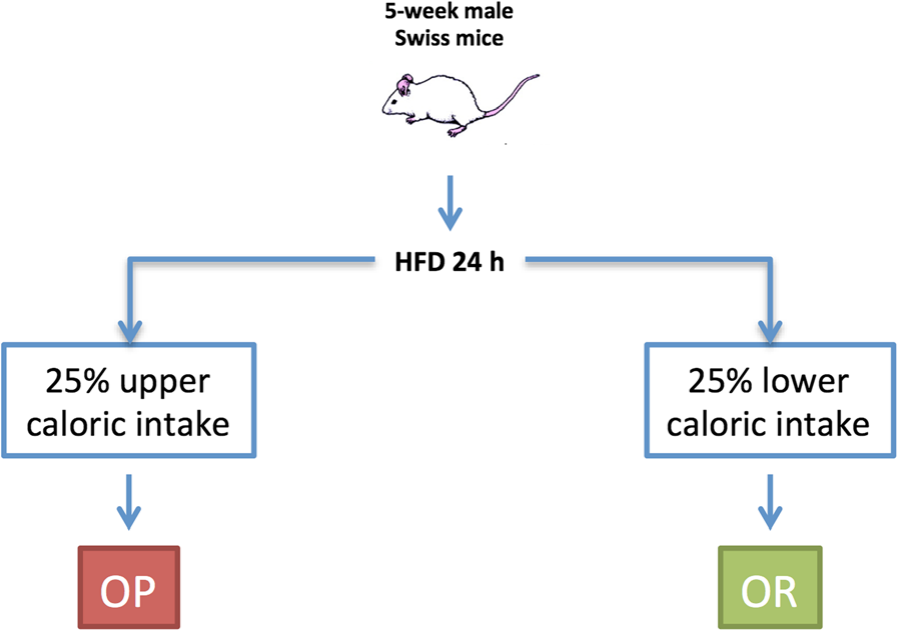
Schematic representation of the protocol employed to define obese-prone and obese-resistant mice. Five-week old male Swiss mice were fed on a high-fat diet (HFD) for 24 h. Total caloric intake was determined and mice were divided into quartiles based on the 24-h caloric intake. Mice on the upper quartile were defined as obese prone (OP) and mice on the lower quartile were defined as obese resistant (OR)

Male Swiss mice were provided by the University of Campinas Breeding Center and the study was approved by the Ethics Committee of the University of Campinas (Project #: CEUA 2926–1). For all experiments, 5-week-old mice were kept in individual cages in a silent environment with controlled temperature using a 12 h light/12 h dark cycle (6/18 h). Mice had free access to water and food ad libitum. For the experiments, mice were randomly divided into groups fed either chow or a HFD (composition of the diets is presented in Additional file 1: Table S1). To identify OP and OR mice, we used a previously described protocol [9]. In short, 8-week-old male Swiss mice were fed a HFD for 24 h, and total caloric intake was recorded. Mice on the upper quartile of caloric intake were defined as OP, whereas mice on the lower quartile of caloric intake were defined as OR (Fig. 1). Thereafter, mice were fed chow for 15 days and the inhibition of LIF was performed. On the next day, HFD was offered for up to 15 days. Body mass and caloric intake were determined every second day. At the end of each experiment, mice were sacrificed by decapitation. For this, they were anesthetized with sodium thiopental (25 mg/kg). Tissue specimens were extracted and stored at −80 °C until analysis.

### Body composition

The body composition was assessed by dual energy X-ray absorptiometry (DEXA, Discovery Wi QDR Series; Hologic Apex Software, Hologic Inc.) in mice fed a HFD for 14 days.

### PCR array

The PCR array was performed after 1 day of HFD in OP or OR mice. The kit used for the determination of gene expression was the Chemokines and Receptors PCR Array (PAMM-022Z, Qiagen). This is a 96-gene panel, with 84 genes for chemokine/chemokine receptors and the remaining for endogenous and positive controls of the PCR reaction (Additional file 1: Table S2).

### Immunoneutralization of LIF in the hypothalamus

OR mice were randomly selected for the treatment with either an immunoneutralizing antibody against LIF or a non-immune IgG. OP mice were always treated with the non-immune IgG. For this, mice were anesthetized with a mixture of ketamine (100 mg/kg) and xylazine (10 mg/kg) and stereotaxically instrumented using a Stoelting stereotaxic apparatus. The OR anti-LIF group received an intracerebroventricular microinjection of 1.0 μl of a solution of an anti-LIF antibody (40 ng/μl) (sc 1336; Santa Cruz Biotechnology, Santa Cruz, CA) in the third ventricle close to the hypothalamus. In the same way, OR IgG or OP IgG mice received 1.0 μl of non-immune IgG. The coordinates were Bregma 0.0 mm; depth 4.5 mm; lateral 0.0 mm to reach the third ventricle. Thereafter, the animals were fed an HFD ad libitum for 1 or 15 days.

### GTT

The glucose tolerance test (GTT) was performed after 12 days on the HFD. In the morning, mice were fasted for 4 h and the blood collection was carried out from the tail to determine glucose levels. A 25% glucose solution was administrated via intraperitoneal injection, and determinations occurred immediately before injection and after 15, 30, 60, 90, and 120 min. The measurements were performed using a portable glucose meter (Optium Xceed - Abbott®). Then, the area under the glucose curve (AUC) was calculated.

### iTT

The insulin-tolerance test (iTT) was performed after 13 days on the HFD. In the morning, mice were fasted for 4 h and the blood collection was carried out from the tail to determine glucose levels. After 4 h of fasting, the iTT test was performed. The blood collection was carried out from the tail to obtain glucose levels. Insulin (1.5 IU/kg body mass) was administrated via intraperitoneal injection and blood glucose was measured immediately before injection and after 5, 10, 15, 20, 25, and 30 min.

### Respirometry

For acclimation, mice were placed in the respirometric chamber 1 day prior to the beginning of the measurements. The O_2_ consumption, CO_2_ production, respiratory quotient (RQ), and spontaneous activity were determined for a period of 24 h in the Gas Analyzer LE405 Gas Analyzer (Panlab - Harvard Appliance, Holliston, MA, USA). The airflow was maintained by Air Supply and Switching (Airlab - Harvard Apparatus, Holliston, MA, USA) and gas analyzer calibrated with known concentrations of O_2_ and CO_2_ (Air Liquid, São Paulo, Brazil). The calculations were performed using the Metabolism 2.2v software.

### Real-time PCR

Reactions were performed using the TaqMan™ System (Applied Biosystems). The gene glyceraldehyde-3-phosphate dehydrogenase (GAPDH) was chosen as the endogenous control for the reaction. The expression of tumor necrosis factor alpha (TNF-α, Mm00443258_m1), interleukin 1 beta (IL-1β, Mm00434228_m1), interleukin 6 (IL-6, Mm00446190_m1), interleukin 10 (IL-10, Mm01288386_m1), Cx3cl1 (Mm00436454_m1), Ccl20 (Mm01268754_m1), Cxcl1 (Mm.PT.5842076891), and LIF (Mm.PT.58.13926050) was quantified in the hypothalamus. Hypothalami were extracted 1 day after the introduction of the HFD in anti-LIF or IgG-treated mice. For the determination of relative transcript expression, real-time PCR reactions were performed in duplicate as follows: 3.0 μl TaqMan Universal PCR Master Mix 2X, 0.25 μl of the primers and probe solution, 2.75 μl water, and 4.0 μl cDNA. The values of relative gene expression were obtained by analyzing the results using 7500 System SDS software (Applied Biosystems).

### Immunohistochemistry

Six-week-old male Swiss mice fed on chow were anesthetized with a mixture of ketamine (100 mg/kg) and xylazine (10 mg/kg) perfused with saline and 4% paraformaldehyde. The brain was totally removed and kept in a 4% paraformaldehyde solution for 24 h, followed by 48 h in a 30% sucrose solution for cryoprotection. Coronal sectioning at a thickness of 20 μm was performed using a cryostat (LEICA Microsystems, CM1860, Buffalo Grove, IL, USA). Sections were rinsed with PBS and blocked in a solution containing 5% normal serum and 0.2% Tween in phosphate buffered saline for 1 h at room temperature followed by incubation at 4 °C overnight with antibodies against LIF (ab113262, rabbit polyclonal, 1:500, ABCAM, Cambridge, UK) or LIF receptor (sc659, rabbit polyclonal, 1:200, Santa Cruz Biotechnology, Inc.) with ionized calcium binding adaptor molecule 1 (IBA-1, sc28530, goat polyclonal, 1:200, Santa Cruz Biotechnology, Inc.) or neuropeptide Y (NPY, sc133080, mouse monoclonal, 1:200, Santa Cruz Biotechnology, Inc.) or POMC (ab32893, goat polyclonal, 1:500, Abcam, Cambridge, UK) or glial fibrillary acidic protein (GFAP, ab4648, mouse monoclonal, 1:500, Abcam, Cambridge, UK) or alpha-MSH (AB5087, sheep polyclonal, 1:500, Millipore, Billerica, Massachusetts, USA) in a blocking buffer (1% bovine serum albumin in PBS-Tween). Next, sections were incubated for 2 h with goat anti-rabbit Cy3 (ab6941, 1:500, ABCAM, Cambridge, UK) or donkey anti-rabbit Alexa 546 and goat anti-mouse FITC or donkey anti-goat FITC. Nuclear staining was obtained using TO-PRO®-3 Iodide ((642/661) T3605, 1:1000, Life Technologies, Carlsbad, CA, EUA) in PBS. Analysis and documentation of the results were performed using a Leica TCS SP5 II confocal fluorescence microscope.

### Immunoblot

LIF and ^Tyr705^phosphor-STAT3 protein expressions were determined in total protein extract samples by immunoblotting using a previously described method [13].

*Statistics analysis.* Results are presented as the mean ± standard error of the mean (SEM). For the comparison of means between two groups, we used Student’s *t* test for independent samples. Linear regression test was utilized to calculate kITT (based on the ITT test). The significance level was set at *p* < 0.05. Graph Pad Prism® was used to analyze the data.

## Results

### Hypothalamic LIF undergoes distinct regulation in OP and OR mice fed a HFD

**Fig. 2:**
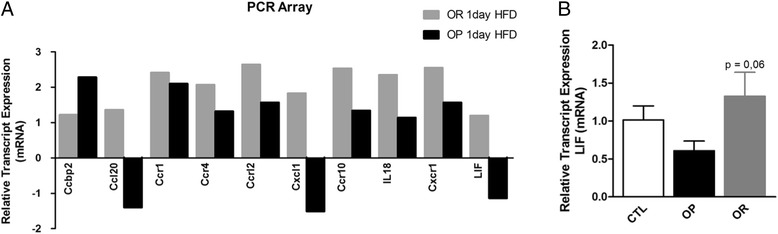
Hypothalamic transcripts differentially expressed between obesity-prone and obesity-resistant mice. **a** The cDNA obtained from hypothalamic RNA was employed in a real-time PCR array to determine the relative transcript expression in mice fed chow or obesity-prone (OP) and obesity-resistant (OR) mice fed on a high-fat diet (HFD) for 1 day; only the transcripts undergoing significant difference from control are presented; the results are presented as transcript expression relative to chow; the identity of the transcripts is presented in Additional file 1: Table S2. **b** The relative expression of LIF transcript was determined by real-time PCR in hypothalamic samples from control (CTL) mice, fed on chow, or OP and OR mice fed on HFD for 1 day. In **a** *n* = 3; in **b** *n* = 5; *p* = 0.06 vs. OP

cDNA obtained from the hypothalamus was employed to evaluate the expression of 84 chemokine-related transcripts in OP and OR mice fed on a HFD for 1 day. The complete list of transcripts evaluated in this study is shown in Additional file 1: Table S2. As a whole, ten transcripts were differentially expressed (Fig. 2a). However, most of them were regulated in the same direction in OP and OR mice. Thus, Ccbp2, Ccr1, Ccr4, Ccrl2, Ccr10, Il18, and Cxcr1 underwent an increase both in OP and OR, as compared to mice fed regular chow (Fig. 2a). Only three transcripts, Ccl20, Cxcl1, and LIF, were differentially regulated in OP and OR mice (Fig. 2a). For all those three transcripts, 1 day of feeding with the HFD was accompanied by an increase in OR and a reduction in OP mice, as compared to mice fed on chow (Fig. 2a). Because of the known involvement of LIF in the control of feeding and regulation of POMC expression in the hypothalamus [12], we decided to further explore the involvement of this protein in the different body compositions, metabolic, and inflammatory phenotypes observed in OP and OR mice fed a HFD. In order to confirm the findings of the real-time PCR array, LIF transcript expression was determined in the hypothalamus of control mice fed chow and in OP and OR mice fed the HFD for 1 day. As depicted in Fig. 2b, LIF transcripts were reduced in the hypothalamus of OP as compared to OR mice.

### LIF and LIF receptor are predominantly co-expressed with NPY and POMC in the hypothalamus of mice

**Fig. 3:**
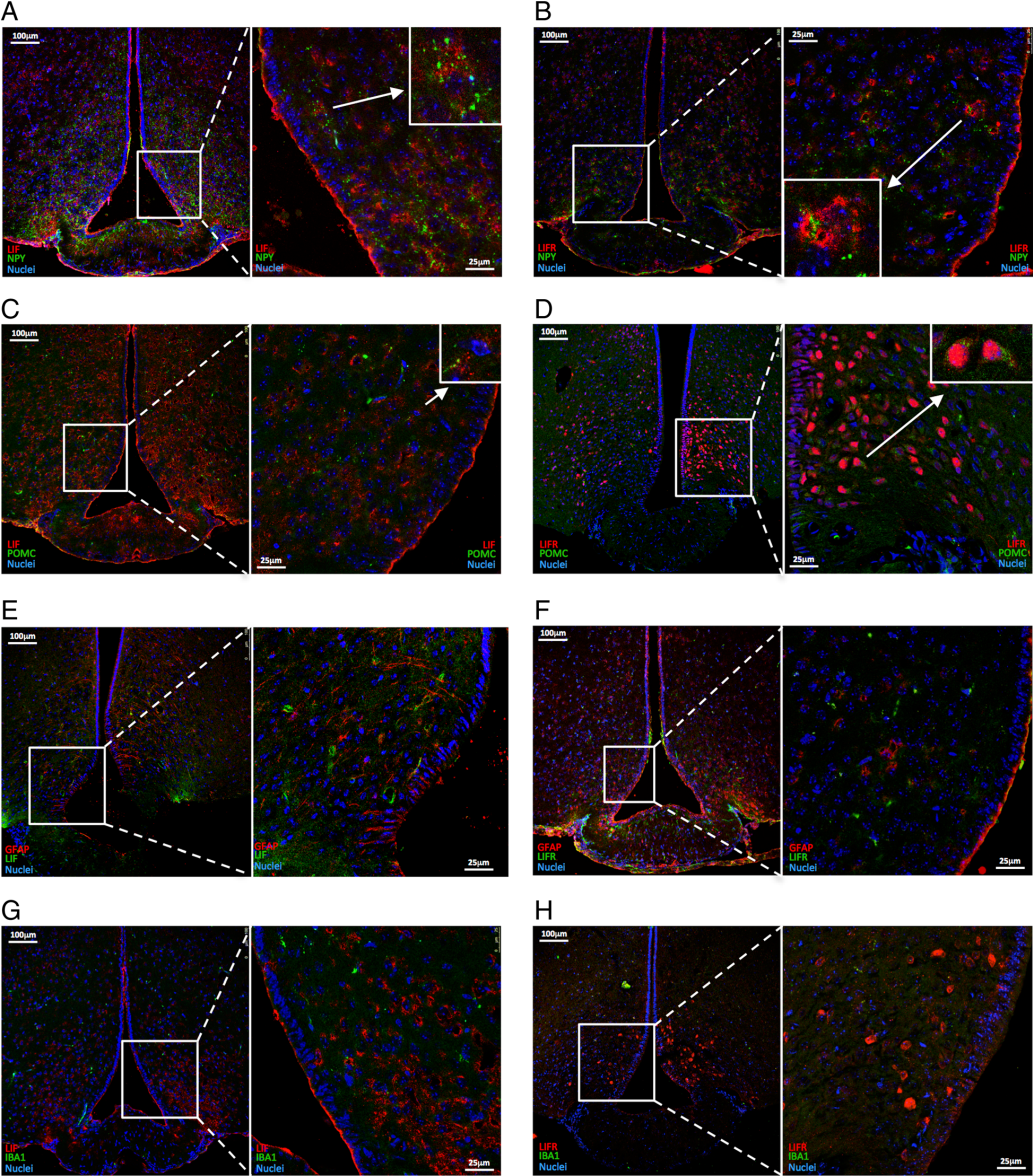
The anatomical and cellular distribution of LIF and LIF receptor in the hypothalamus of mice. Hypothalamic frozen sections (5.0 μm thick) were prepared from 5-week-old mice fed on chow. Immunofluorescence staining was performed using antibodies against LIF (**a**, **c**, **e**, **g**), LIF receptor (LIFR) (**b**, **d**, **f**, **h**), NPY (**a**, **b**), POMC (**c**, **d**), GFAP (**e**, **f**), and IBA1 (**g**, **h**). Nuclei was labeled using DAPI. In some panels, a high magnification image depicts details of cells (arrows). Color code and magnifications are presented in the panels. Figures are representative of three independent experiments

Immunofluorescence staining was employed to determine the anatomical and cellular distribution of LIF and LIF receptor (LIFR) in the hypothalamus of mice. As shown in Fig. 3a–d, most immunostaining of LIF and LIFR co-localize with NPY (Fig. 3a, b) and POMC (Fig. 3c, d). The LIF/LIFR-POMC co-localization was further confirmed by employing anti-alpha-MSH antibody (Additional file 1: Figure S1). Some cells expressing GFAP (astrocytes) also express LIF (Fig. 3e). Virtually no co-localization was detected for LIFR and GFAP (Fig. 3f). In addition, no localization was detected for either LIF or LIFR in cells immunostained with IBA1 (microglia) (Fig. 3g, h).

### Inhibition of hypothalamic LIF in OR mice is accompanied by increased diet-induced hypothalamic inflammation

**Fig. 4:**
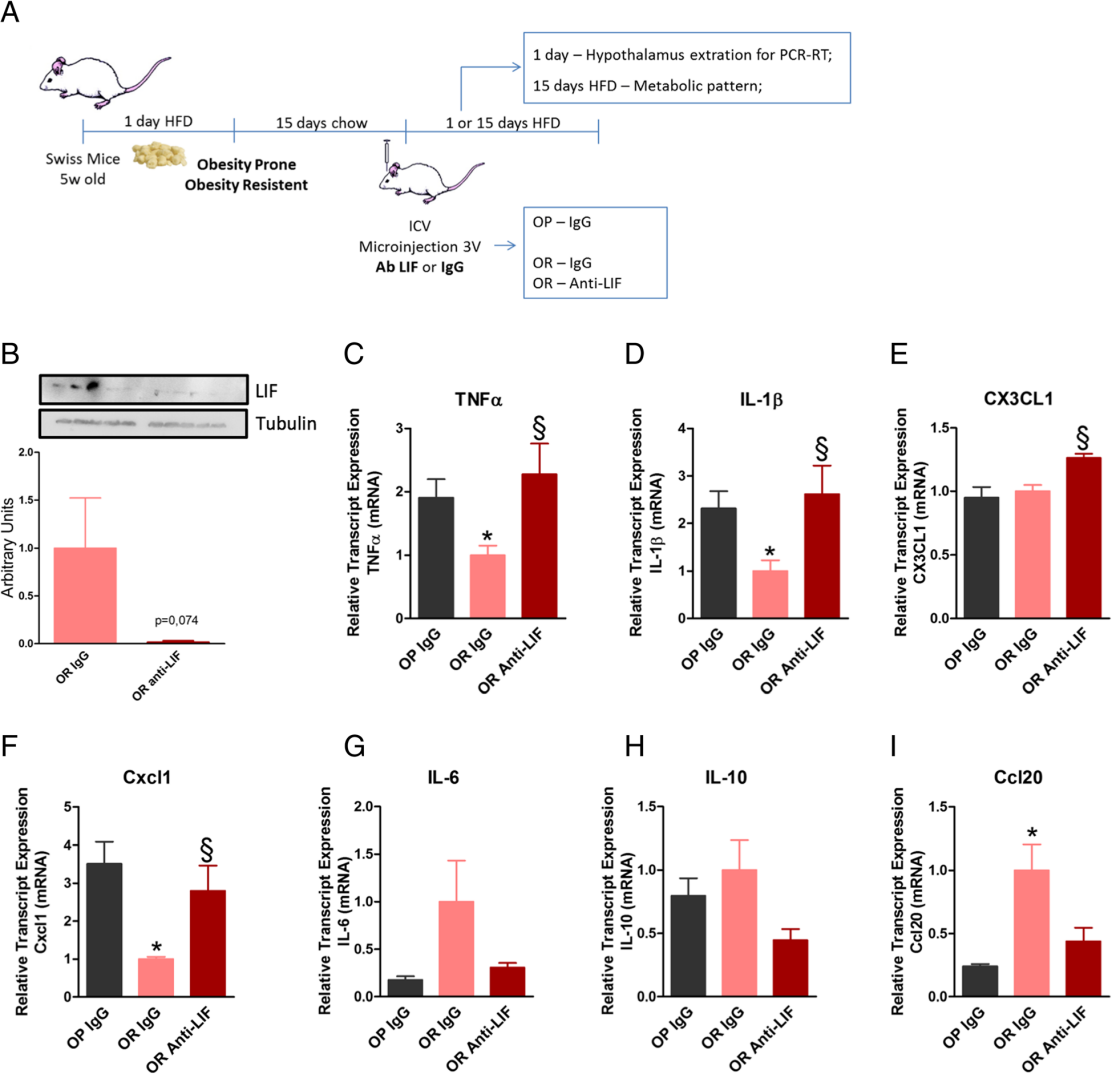
Impact of inhibiting hypothalamic LIF on inflammatory markers. **a** Schematic representation of the protocol employed to immunoneutralize hypothalamic LIF. **b** The expression of LIF protein was evaluated by immunoblot in the hypothalamus of obesity-resistant (OR) mice treated with non-immune IgG or anti-LIF antibody for 15 days. Real-time PCR was employed to determine the relative expression of transcripts encoding for TNF-α (**c**), IL-1β (**d**), CX3CL1 (fractalkine) (**e**), cxcl1 (**f**) IL-6 (**g**), and IL-10 (**h**) ccl20 (**i**) in the hypothalamus of obesity-prone (OP) mice treated with non-immune IgG or OR mice treated either with non-immune IgG or anti-LIF antibody. In **b**–**g**, *n* = 5; *p < 0.05 vs. OP IgG and §*p* < 0.05 vs. OR IgG

Because LIF expression was rapidly induced in the hypothalamus of OR mice fed a HFD, we hypothesized that it could play a protective role against the progression to diet-induced obesity. In order to test this hypothesis, mice were submitted to a protocol as depicted in Fig. 4a, which was designed to successfully immunoneutralize hypothalamic LIF, as shown by immunoblot (Fig. 4b); thereafter, a number of inflammatory and metabolic parameters were evaluated. The immunoneutralization of hypothalamic LIF resulted in an early increase in the expression of transcripts of inflammatory proteins, TNF-α (Fig. 4c), IL1β (Fig. 4d), Cx3cl1 (Fig. 4e), and Cxcl1 (Fig. 4f). This was accompanied by a trend to reduction of the transcripts of the anti-inflammatory proteins, IL6 (Fig. 4g), IL10 (Fig. 4h), and Ccl20 (Fig. 4i). There were no changes in the expression of transcript encoding for NPY and POMC (data not shown). The immunoneutralization of LIF in the hypothalamus resulted in reduced STAT3 Tyr705 phosphorylation in the hypothalamus but not in the hippocampus [14] (Additional file 1: Figure S2), suggesting that the method was appropriate for a site-specific action of the antibody.

### Inhibition of hypothalamic LIF transforms OR in OP mice

**Fig. 5:**
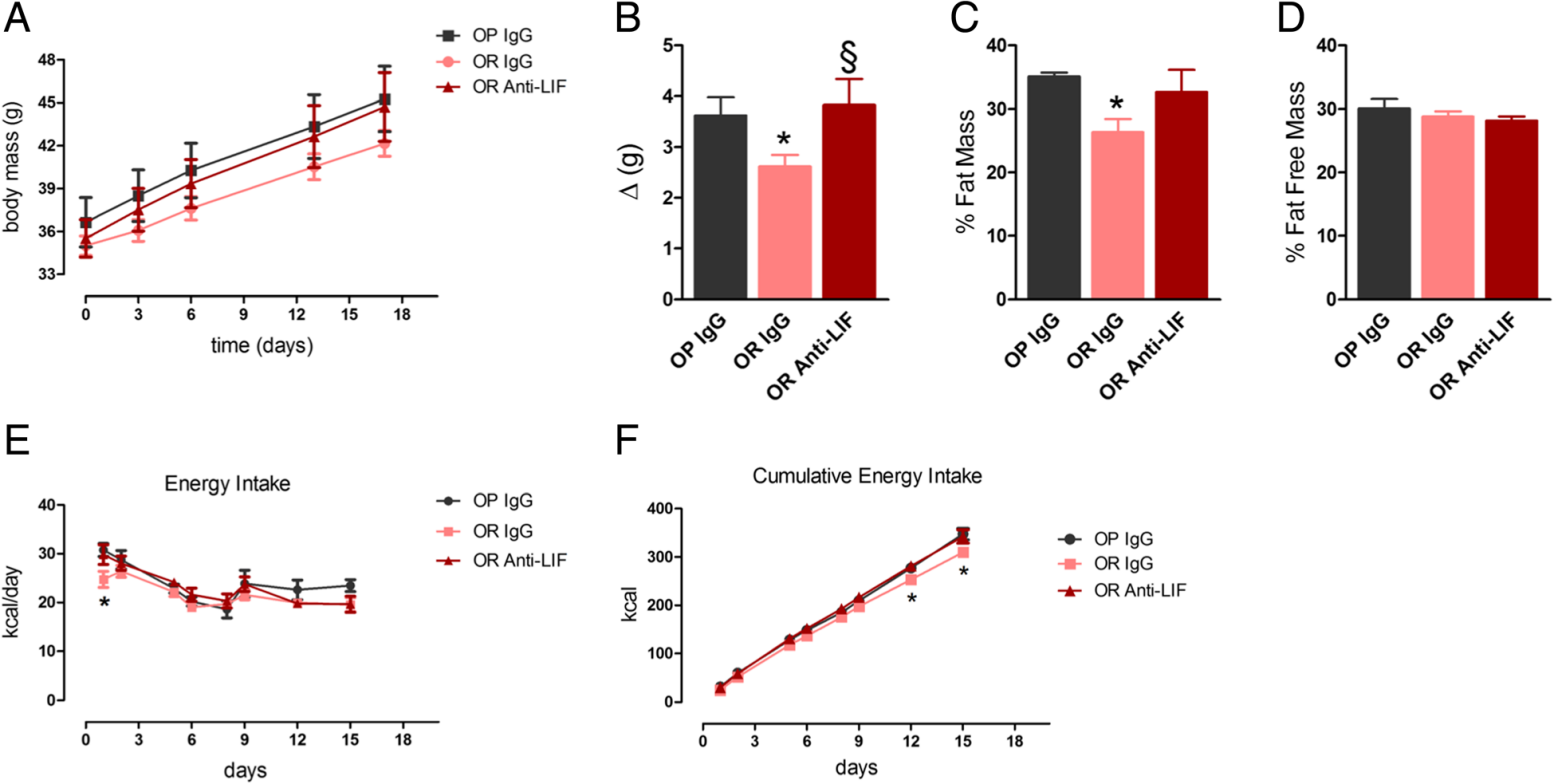
Body mass, body composition, and caloric intake in mice treated with anti-LIF antibody. Mice were treated according to the protocol presented in Fig. 3a, and parameters were determined throughout the experimental period. **a** Body mass and **b** body mass variation. **c** Relative fat mass and **d** relative fat-free mass, as determined by body densitometry at the end of the experimental period. **e** Daily energy intake and **f** cumulative energy intake during the experimental period. In all experiments *n* = 5; *p < 0.05 vs. OP IgG and §*p* < 0.05 vs. OR IgG. OP IgG, obesity-prone mice treated with non-immune IgG; OR IgG, obesity-resistant mice treated with non-immune IgG; OR anti-LIF, obesity-resistant mice treated with anti-LIF antibody

**Fig. 6:**
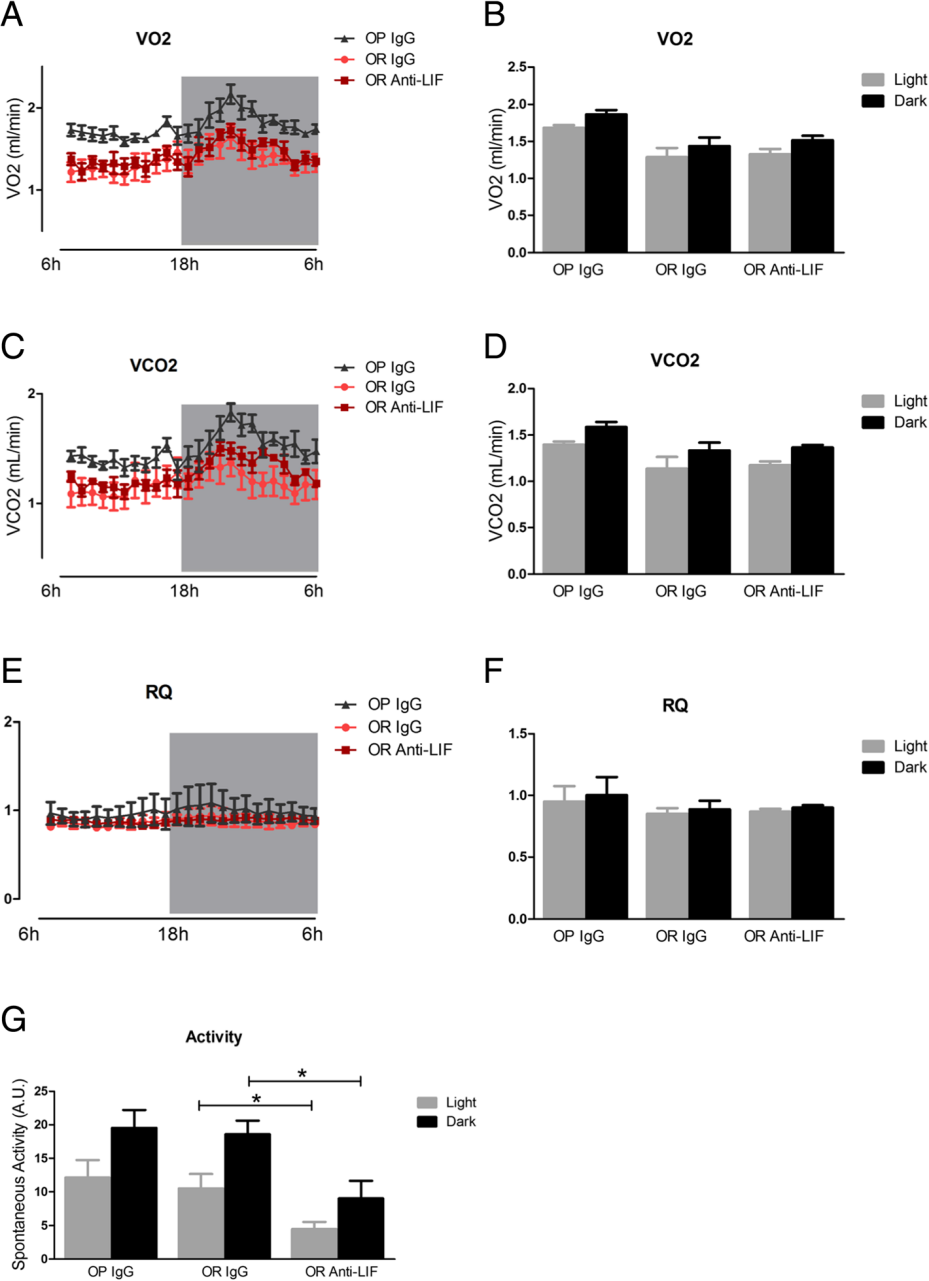
Respirometry and spontaneous activity in mice treated with anti-LIF antibody. Mice were treated according to the protocol presented in Fig. 3a and parameters were determined at the end of the experimental period. In respirometry, vO_2_ (**a**, **b**) and vCO_2_ (**c**, **d**) were measured during a 24-h period (**a**, **c**) and the means during light and dark periods were calculated (**b**, **d**). The respiratory quotient (RQ) was calculated during the 24-h period (**e**) and means were obtained during the light and dark periods (**f**). Spontaneous activity was determined during a 24-h period (**g**). In all experiments *n* = 5; *p < 0.05. OP IgG, obesity-prone mice treated with non-immune IgG; OR IgG, obesity-resistant mice treated with non-immune IgG; OR anti-LIF, obesity-resistant mice treated with anti-LIF antibody

The immunoneutralization of hypothalamic LIF in OR mice resulted in increased body mass gain over a period of 15 days (Fig. 5a, b). The change in body mass was mostly due to an increase in fat mass (Fig. 5c, d). To explore the mechanisms involved in the phenotypic change in OR mice, we evaluated caloric intake, energy expenditure and spontaneous physical activity. As shown in Fig. 5e, daily caloric intake was similar during most of the experimental period; only on the first day was there a significant increase in the caloric consumption of OR mice treated with the anti-LIF antibody. However, during the 15-day experimental period, there was a significant increase in cumulative caloric intake in OR mice treated with the anti-LIF antibody (Fig. 5f), which resulted in caloric intake similar to OP mice. The immunoneutralization of hypothalamic LIF promoted no changes in O_2_ consumption, CO_2_ production, or the respiratory quotient (Fig. 6a–f). However, OR mice treated with the anti-LIF antibody presented significantly reduced spontaneous physical activity during both the light and dark cycles (Fig. 6g).

### Inhibition of hypothalamic LIF induces glucose intolerance in OR mice

**Fig. 7:**
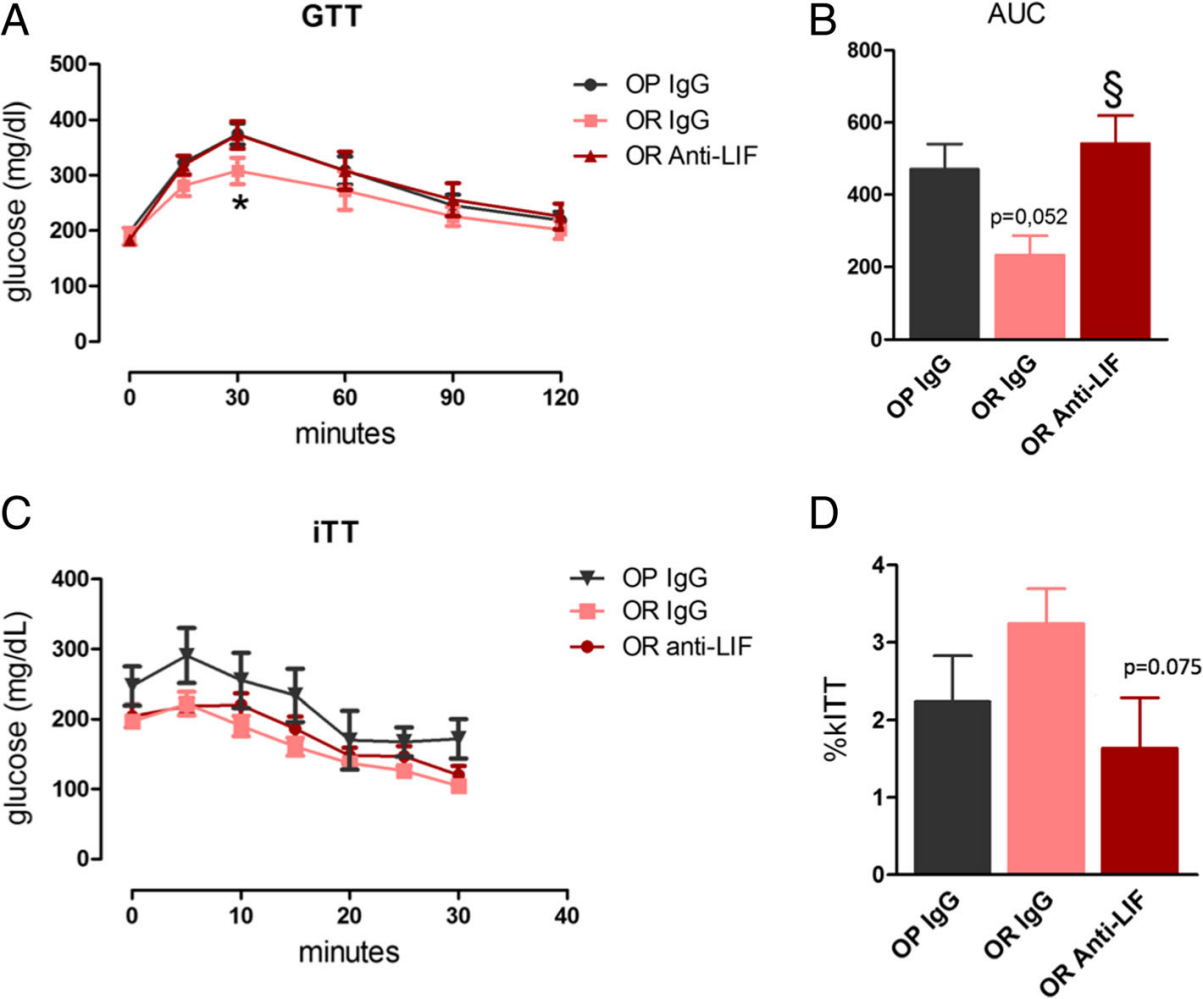
Determination of glucose tolerance in mice treated with anti-LIF antibody. Mice were treated according to the protocol presented in Fig. 3a and parameters were determined at the end of the experimental period. In the glucose tolerance test (GTT), blood glucose variation was measured from time 0 to 120 min (**a**) and the area under the glucose curve (AUC) was calculated (**b**). In insulin-tolerance test (ITT) blood glucose variation was measured form time 0 to 30 min (**c**) and the constant for glucose decay (kITT) was calculated (**d**). In all experiments *n* = 5; *p < 0.05 vs. OP IgG and §*p* < 0.05 vs. OR IgG. In **b**, *p* = 0.052 vs. OP IgG; in **d**, *p* = 0.075 vs. OR IgG. OP IgG, obesity-prone mice treated with non-immune IgG; OR IgG, obesity-resistant mice treated with non-immune IgG; OR anti-LIF, obesity-resistant mice treated with anti-LIF antibody

We have previously shown that, in contrast to OP mice, glucose tolerance is preserved in OR mice fed an HFD [9]. Here, we evaluated glucose tolerance in OR mice treated with the anti-LIF antibody in the hypothalamus. As shown in Fig. 7a, the immunoneutralization of hypothalamic LIF resulted in higher blood glucose levels during a glucose tolerance test, leading to an increased area under the glucose curve (Fig. 7b). At least in part, the deterioration in glucose tolerance in OR mice treated with the anti-LIF antibody was due to the development of insulin resistance, as determined by an insulin-tolerance test (Fig. 7c) and the determination of the constant for glucose decay during the insulin tolerance test (kITT) (Fig. 7d).

### Increased caloric intake explains most of the phenotypic change in OR mice treated with the anti-LIF antibody

**Fig. 8:**
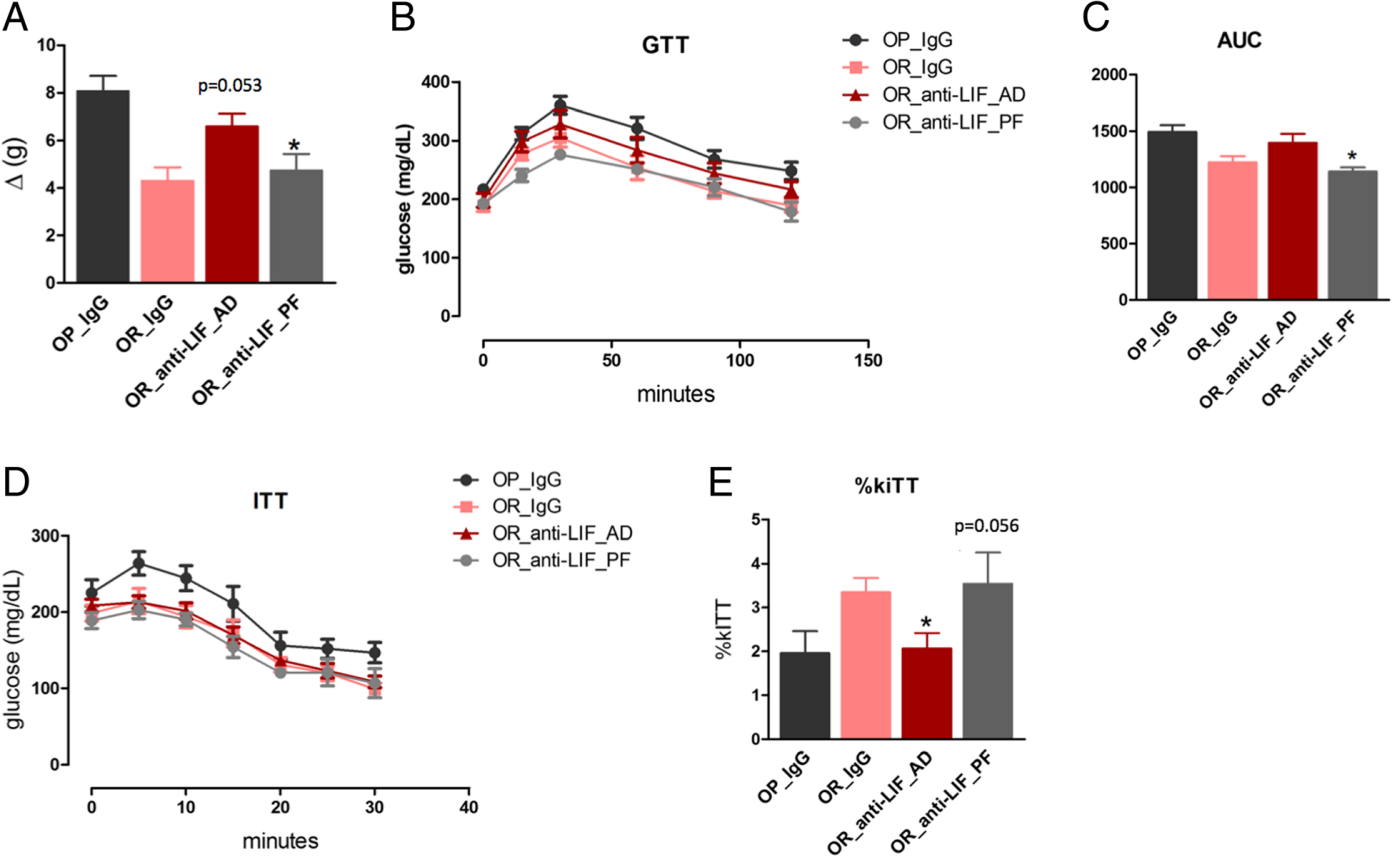
The impact of pair feeding on the phenotype of obesity-resistant mice treated with anti-LIF antibody*.* Mice were treated with a protocol similar to the one presented in Fig. 3a, except that a group was submitted to pair feeding with the obesity-resistant mice treated with IgG throughout the experimental period. At the end of the experimental period, body mass variation was determined (**a**). In the glucose tolerance test (GTT), blood glucose variation was measured from time 0 to 120 min (**b**) and the area under the glucose curve (AUC) was calculated (**c**). In insulin-tolerance test (ITT), blood glucose variation was measured form time 0 to 30 min (**d**) and the constant for glucose decay (kITT) was calculated (**e**). In all experiments *n* = 5. In **a**, *p* = 0.053 vs. OR IgG; *p < 0.05 vs. OR anti-LIF AD. In **c**, *p < 0.05 vs. OR anti-LIF AD. In **e**, *p < 0.05 vs. OR IgG; *p* = 0.056 vs. OR anti-LIF AD

In order to test the hypothesis that the OP-like phenotype developed by OR mice treated with the anti-LIF antibody was due to increased caloric intake (as shown in Fig. 5f), we performed an experiment in which OR mice treated with the anti-LIF antibody were fed a similar caloric amount consumed spontaneously by OR mice treated with IgG. As shown in Fig. 8a, pair feeding was sufficient to normalize body mass gain. This was accompanied by the restoration of glucose tolerance (Fig. 8b, c) and also by the restoration of insulin activity (Fig. 8d, e).

## Discussion

Diet-induced hypothalamic inflammation plays an important role in the development of obesity in a number of experimental models [15, 16]. The mechanistic link between hypothalamic inflammation and obesity is illustrated by the fact that several approaches that target inflammatory pathways in the hypothalamus of obese rodents result in the attenuation of the obese phenotype and invariably in the improvement of distinct aspects of the obesity-associated phenotypes, such as insulin resistance, diabetes, and hypertension [2, 3, 17–21]. Due to anatomical constraints, only a few studies have evaluated the hypothalamus of obese humans [18, 22, 23]. Magnetic resonance imaging is capable of detecting both functional and structural abnormalities in the hypothalamus of obese subjects, which could be, at least in part, reverted following body mass reduction [22, 23]. Unfortunately, for most people, medical attention occurs in the late phases of obesity, and both human and experimental studies have shown that neuronal loss and gliosis are present at this stage, suggesting that complete restoration of hypothalamic physiology in the control of body mass may be a difficult task [16, 18, 22, 23]. Nevertheless, understanding the mechanisms involved in the early damage to the hypothalamus in obesity may provide new strategies to prevent the development of this threatening condition. With this concept in mind, we decided to evaluate the very early inflammatory events occurring in the hypothalamus of mice fed an HFD.

In a previous study [9], we have shown that outbred mice fed a HFD present a normal distribution of body mass gain. Mice gaining weight in the upper quartile are OP and present a high predisposition for the development of glucose intolerance, whereas mice in the lower quartile are OR. This provides an interesting experimental model that reproduces the human predisposition to obesity. In the first part of the study, we asked if, after 1 day on an HFD, OP and OR mice would present different expression of transcripts encoding for proteins related to chemokines. In fact, out of 84 transcripts evaluated, ten (12%) presented some sort of modulation in response to the diet. However, in most cases, the variation in expression was similar in OP and OR mice. Only three transcripts, encoding for Ccl20, Cxcl1 and LIF, were differentially regulated in OP and OR mice. For these three transcripts, the expression was increased in OR and reduced in OP mice.

Ccl20 encodes for the CCL20 protein, also known as liver activation-regulated chemokine (LARC) [24]. Its expression can be induced by microbial factors, LPS, TNF-α, and IFN-γ and has been reported to occur in several tissues and cell types, predominating in lymphocytes, lymphoid tissues, and liver [25, 26]. No previous study has reported the expression of Ccl20 in the hypothalamus; however, one study has shown than circulating CCL20 is reduced in mice chronically fed an HFD, suggesting that it is somehow affected by dietary factors [27].

Cxcl1 encodes for the CXCL1 protein, also known as neutrophil-activating protein 3 (NAP3) and melanoma growth stimulating activity alpha (MSGAa) [28]. It is expressed by epithelial cells, neutrophils, and macrophages and has neutrophil chemoattractant activity [29]. In the central nervous system, it has been shown to inhibit the migration of oligodendrocyte precursors [30]. A number of studies have reported the expression and involvement of CXCL1 in distinct processes regulated by the hypothalamus [31, 32]; however, only one study showed that the systemic expression of CXCL1 is increased in response to dietary fats [27].

Out of the three transcripts of interest for the main purpose of this study, LIF was undisputedly the one with richest data published regarding its potential involvement in the hypothalamic control of caloric intake and energy expenditure. Studies published during the early 1990s reported that LIF can be produced by cancer cells and induce cachexia [33, 34]. The initial suspicion was that it acted mostly in peripheral tissues, such as the adipose tissue, to induce the wastage syndrome in cancer [34, 35]. During the late 1990s, other lines of investigation led to the demonstration of the involvement of LIF in the regulation of POMC. It was shown that, in corticotrophs, LIF could regulate POMC through a mechanism dependent on STAT1 and STAT3 signaling [36]. However, the earliest studies were mostly focused on the impact of LIF on the POMC-dependent regulation of pituitary function [37–39]. Only in 1999 was it proposed that the involvement of LIF in the regulation of hypothalamic neurotransmitters could mediate its cachexia-inducing effects [40]. Moreover, some studies have shown that LIF can also modulate NPY, another important hypothalamic neurotransmitter involved in the regulation of caloric intake and energy expenditure [41, 42]. Thereafter, a number of studies explored the hypothalamic actions of LIF in cachexia [11, 41, 43, 44].

Despite the extensive work performed in the evaluation of LIF in cachexia and the regulation of the pituitary function, no previous study has evaluated the role of hypothalamic LIF in obesity. Here, we hypothesized that the distinct early regulation of hypothalamic LIF in OP and OR mice could explain at least in part the differences in the phenotypes. To test this hypothesis, we inhibited hypothalamic LIF and evaluated a number of metabolic and inflammatory parameters.

The inhibition of hypothalamic LIF in OR mice was sufficient to promote a complete shift on their phenotype transforming the OR in OP mice. As a rule, changes in adiposity and body mass are usually due to modification in caloric intake, energy expenditure, or spontaneous physical activity, or yet, a combination of some of these parameters [45]. In the LIF-inhibited mice, there were changes in cumulative caloric intake and in the physical activity. However, when we performed pair-feeding experiments, there was an almost complete restoration of the original OR phenotype, suggesting that most of the phenotype change promoted by the hypothalamic inhibition of LIF was due to cumulative caloric intake. Because of that, it could be expected that inhibition of LIF might result in changes in the expression of hypothalamic neurotransmitters involved in the regulation of feeding. However, there were no changes in the expression of POMC and NPY, two of the most important neurotransmitters expressed by first order neurons of the arcuate nucleus. We propose at least two scenarios that could explain this apparent dissociation between neuropeptide transcript regulation and the resulting phenotype: (i) the post-transcriptional processing of POMC could be resulting in increased production of anorexigenic a-MSH instead of orexigenic b-endorphin; (ii) neurotransmitter regulation could be taking place not in first order neurons but in neurons that are downstream of the main energy homeostasis neurocircuits.

Another remarkable outcome of the inhibition of hypothalamic LIF was the generation of glucose intolerance. Both the GTT and the ITT revealed a worsening of body glucose handling, which suggests that OR mice under hypothalamic LIF inhibition develop systemic insulin resistance. In the pair-feeding experiment, both GTT and ITT were restored to normality, so we believe that changes in body mass could be the main factor responsible for the abnormality in glucose tolerance [45]. However, studies have shown that defective activity of hypothalamic neurons, both in obesity and aging, can worsen glucose tolerance due to changes in neural output to the liver [19, 21]. Although we did not test this hypothesis in the present work, this is a possibility that cannot be discarded.

## Conclusion

Hypothalamic LIF has emerged as an inflammatory protein that undergoes rapid modulation in response to the consumption of large amounts of dietary fats. Its regulation occurs in opposite directions in mice that are OP and OR, suggesting that it may play a role in these different phenotypes. When hypothalamic LIF is inhibited in OR mice, there is a complete shift of the phenotype, transforming OR into OP mice. Hypothalamic LIF may be an important target to prevent the development of diet-induced obesity.

## Additional file

Additional file 1: Figure S1. The anatomical and cellular distribution of LIF and LIF receptor in the hypothalamus of mice. Hypothalamic 5.0 μm frozen sections were prepared from 5-week old mice fed on chow. Immunofluorescence staining was performed using antibodies against LIF (A), LIF receptor (LIFR) (B), alpha-MSH (aMSH) (A, B). The high magnification images depict details of cells (arrows). Nuclei was labeled using DAPI. Color code and magnifications are presented in the panels. Figures are representative of three independent experiments. **Figure S2. **The impact of LIF immunoneutralization on STAT3 phosphorylation in the hippocampus and hypothalamus. The expression of phosphor-STAT3 (pSTAT3) protein was evaluated by immunoblot in the hippocampus (A) and hypothalamus (B) of obesity-resistant mice treated with non-immune IgG or anti-LIF antibody for 15 days. *N* = 3–4; **p* < 0.05 vs. IgG. Table S1. Composition of the diets. Table S2. Transcripts evaluated with the real-time PCR array. (PDF 18097 kb)

## Abbreviations

CX3CL1: Fractalkine
GAPDH: Glyceraldehyde-3-phosphate dehydrogenase
GFAP: Glial fibrillary acidic protein
GTT: Glucose tolerance test
HFD: High-fat diet
Iba1: Ionized calcium binding adaptor molecule 1
IL-10: Interleukin 10
IL-1β: Interleukin 1 beta
IL-6: Interleukin 6
iTT: Insulin tolerance test
LIF: Leukemia inhibitory factor
NPY: Neuropeptide Y
OP: Obesity prone
OR: Obesity resistant
POMC: Proopiomelanocortin
RQ: Respiratory quotient
TNF-α: Tumor necrosis factor alpha
