# Supplementary material for: Inhibition of hypothalamic leukemia inhibitory factor exacerbates diet-induced obesity phenotype
Source: J Neuroinflammation. 2017 Sep 2;14:178. doi: 10.1186/s12974-017-0956-9 (PMC5581454; doi:10.1186/s12974-017-0956-9)
Supplement: Additional file 1: Figure S1. — The anatomical and cellular distribution of LIF and LIF receptor in the hypothalamus of mice. Hypothalamic 5.0 μm frozen sections were prepared from 5-week old mice fed on chow. Immunofluorescence staining was performed using antibodies against LIF (A), LIF receptor (LIFR) (B), alpha-MSH (aMSH) (A, B). The high magnification images depict details of cells (arrows). Nuclei was labeled using DAPI. Color code and magnifications are presented in the panels. Figures are representative of three independent experiments. Figure S2. The impact of LIF immunoneutralization on STAT3 phosphorylation in the hippocampus and hypothalamus. The expression of phosphor-STAT3 (pSTAT3) protein was evaluated by immunoblot in the hippocampus (A) and hypothalamus (B) of obesity-resistant mice treated with non-immune IgG or anti-LIF antibody for 15 days. N = 3–4; *p < 0.05 vs. IgG. Table S1. Composition of the diets. Table S2. Transcripts evaluated with the real-time PCR array. (PDF 18097 kb) [file 12974_2017_956_MOESM1_ESM.pdf]

Additional file

Inhibition of hypothalamic leukemia inhibitory factor exacerbates diet-induced obesity phenotype

Milena Fioravante[1], Bruna Bombassaro[1], Albina F. Ramalho[1], Nathalia R. Dragano[1], Joseane Morari[1], Carina Solon[1], Natalia Tobar[2], Celso D. Ramos[2], Licio A. Velloso[1]

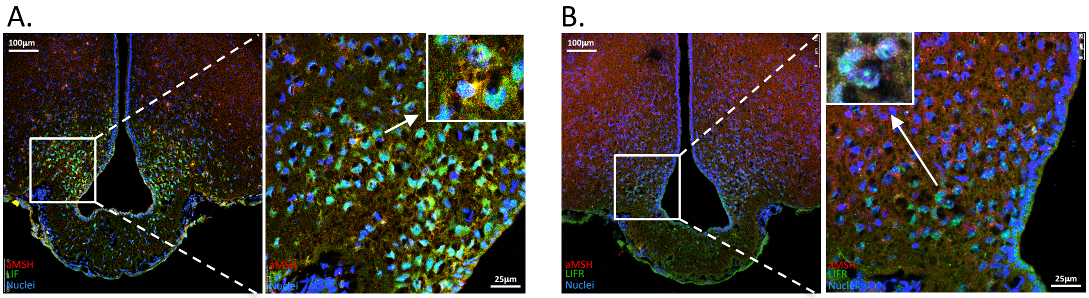

Figure S1 . *The anatomical and cellular distribution of LIF and LIF receptor in the hypothalamus of mice.* Hypothalamic 5.0μm frozen sections were prepared from 5-week old mice fed on chow. Immunofluorescence staining was performed using antibodies against LIF (A), LIF receptor (LIFR) (B), alpha-MSH (aMSH) (A, B). The high magnification images depict details of cells (arrows). Nuclei was labeled using DAPI. Color code and magnifications are presented in the panels. Figures are representative of three independent experiments.

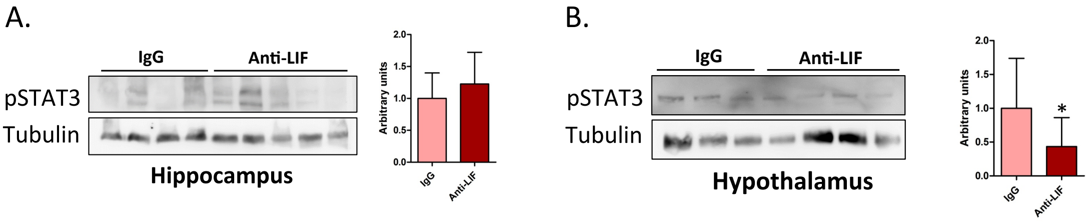

Figure S2 . *The impact of LIF immunoneutralization on STAT3 phosphorylation in the hippocampus and hypothalamus.* The expression of phosphor-STAT3 (pSTAT3) protein was evaluated by immunoblot in the hippocampus (A) and hypothalamus (B) of obesity-resistant mice treated with non-immune IgG or anti-LIF antibody for 15 days. N=3-4; *p<0.05 vs. IgG.

Table S1. *Composition of the diets*

Ingredients	Chow - Control (g)	High Fat Diet (g)
Starch	427,5	115,5
Protein (Casein)	200	200
Dextrinizated Corn Starch	132	132
Sucrose	100	100
Soybean Oil	40	40
Lard	0	312
L-Cystine	3	3
Choline	2,5	2,5
Cellulose (Fiber)	50	50
Minerals Mix	35	35
Vitamins Mix	10	10
**Total**	1000	1000

Table S2. *Transcripts evaluated with the real-time PR array*

Uni Gene	Ref Seq	Symbol	Description
Mm.29368	NM_011784	Aplnr	Apelin receptor
Mm.1442	NM_007540	Bdnf	Brain derived neurotrophic factor
Mm.6246	NM_007551	Cxcr5	Chemokine (C-X-C motif) receptor 5
Mm.483321	NM_009756	Bmp10	Bone morphogenetic protein 10
Mm.42160	NM_009757	Bmp15	Bone morphogenetic protein 15
Mm.385759	NM_007556	Bmp6	Bone morphogenetic protein 6
Mm.258105	NM_021609	Ccbp2	Chemokine binding protein 2
Mm.1283	NM_011329	Ccl1	Chemokine (C-C motif) ligand 1
Mm.4686	NM_011330	Ccl11	Chemokine (C-C motif) ligand 11
Mm.867	NM_011331	Ccl12	Chemokine (C-C motif) ligand 12
Mm.41988	NM_011332	Ccl17	Chemokine (C-C motif) ligand 17
Mm.424740	NM_011888	Ccl19	Chemokine (C-C motif) ligand 19
Mm.290320	NM_011333	Ccl2	Chemokine (C-C motif) ligand 2
Mm.116739	NM_016960	Ccl20	Chemokine (C-C motif) ligand 20
Mm.244263	NM_013652	Ccl4	Chemokine (C-C motif) ligand 4
Mm.284248	NM_013653	Ccl5	Chemokine (C-C motif) ligand 5
Mm.137	NM_009139	Ccl6	Chemokine (C-C motif) ligand 6
Mm.341574	NM_013654	Ccl7	Chemokine (C-C motif) ligand 7
Mm.42029	NM_021443	Ccl8	Chemokine (C-C motif) ligand 8
Mm.416125	NM_011338	Ccl9	Chemokine (C-C motif) ligand 9
Mm.274927	NM_009912	Ccr1	Chemokine (C-C motif) receptor 1
Mm.423532	NM_007718	Ccr1l1	Chemokine (C-C motif) receptor 1-like 1
Mm.6272	NM_009915	Ccr2	Chemokine (C-C motif) receptor 2

Mm.57050	NM_009914	Ccr3	Chemokine (C-C motif) receptor 3
Mm.1337	NM_009916	Ccr4	Chemokine (C-C motif) receptor 4
Mm.14302	NM_009917	Ccr5	Chemokine (C-C motif) receptor 5
Mm.8007	NM_009835	Ccr6	Chemokine (C-C motif) receptor 6
Mm.2932	NM_007719	Ccr7	Chemokine (C-C motif) receptor 7
Mm.442098	NM_007720	Ccr8	Chemokine (C-C motif) receptor 8
Mm.440604	NM_009913	Ccr9	Chemokine (C-C motif) receptor 9
Mm.269254	NM_145700	Ccrl1	Chemokine (C-C motif) receptor-like 1
Mm.7336	NM_017466	Ccrl2	Chemokine (C-C motif) receptor-like 2
Mm.272746	NM_027022	Cmtm2a	CKLF-like MARVEL transmembrane domain containing 2A
Mm.390108	NM_024217	Cmtm3	CKLF-like MARVEL transmembrane domain containing 3
Mm.383258	NM_153582	Cmtm4	CKLF-like MARVEL transmembrane domain containing 4
Mm.41614	NM_026066	Cmtm5	CKLF-like MARVEL transmembrane domain containing 5
Mm.28858	NM_026036	Cmtm6	CKLF-like MARVEL transmembrane domain containing 6
Mm.5196	NM_008153	Cmklr1	Chemokine-like receptor 1
Mm.6522	NM_007722	Cxcr7	Chemokine (C-X-C motif) receptor 7
Mm.795	NM_007778	Csf1	Colony stimulating factor 1 (macrophage)
Mm.4922	NM_009969	Csf2	Colony stimulating factor 2 (granulocyte-macrophage)
Mm.103711	NM_009142	Cx3cl1	Chemokine (C-X3-C motif) ligand 1
Mm.44065	NM_009987	Cx3cr1	Chemokine (C-X3-C) receptor 1
Mm.21013	NM_008176	Cxcl1	Chemokine (C-X-C motif) ligand 1
Mm.877	NM_021274	Cxcl10	Chemokine (C-X-C motif) ligand 10
Mm.131723	NM_019494	Cxcl11	Chemokine (C-X-C motif) ligand 11
Mm.303231	NM_021704	Cxcl12	Chemokine (C-X-C motif) ligand 12
Mm.10116	NM_018866	Cxcl13	Chemokine (C-X-C motif) ligand 13
Mm.64326	NM_011339	Cxcl15	Chemokine (C-X-C motif) ligand 15
Mm.4979	NM_009140	Cxcl2	Chemokine (C-X-C motif) ligand 2
Mm.332490	NM_019932	Pf4	Platelet factor 4
Mm.4660	NM_009141	Cxcl5	Chemokine (C-X-C motif) ligand 5
Mm.293614	NM_023785	Ppbp	Pro-platelet basic protein
Mm.766	NM_008599	Cxcl9	Chemokine (C-X-C motif) ligand 9
Mm.12876	NM_009910	Cxcr3	Chemokine (C-X-C motif) receptor 3
Mm.1401	NM_009911	Cxcr4	Chemokine (C-X-C motif) receptor 4
Mm.124289	NM_030712	Cxcr6	Chemokine (C-X-C motif) receptor 6
Mm.287977	NM_138302	Tymp	Thymidine phosphorylase
Mm.4744	NM_008109	Gdf5	Growth differentiation factor 5
Mm.8021	NM_007721	Ccr10	Chemokine (C-C motif) receptor 10
Mm.33362	NM_175520	Gpr81	G protein-coupled receptor 81
Mm.3879	NM_010431	Hif1a	Hypoxia inducible factor 1, alpha subunit
Mm.1284	NM_008355	Il13	Interleukin 13
Mm.10137	NM_010551	Il16	Interleukin 16
Mm.1410	NM_008360	Il18	Interleukin 18
Mm.15534	NM_010554	Il1a	Interleukin 1 alpha
Mm.276360	NM_021283	Il4	Interleukin 4
Mm.337035	NM_178241	Cxcr1	Chemokine (C-X-C motif) receptor 1
Mm.234466	NM_009909	Cxcr2	Chemokine (C-X-C motif) receptor 2
Mm.1100	NM_010564	Inha	Inhibin alpha
Mm.3092	NM_008381	Inhbb	Inhibin beta-B
Mm.4964	NM_008501	Lif	Leukemia inhibitory factor

Mm.159670	NM_020490	Ltb4r2	Leukotriene B4 receptor 2
Mm.29564	NM_008610	Mmp2	Matrix metallopeptidase 2
Mm.213003	NM_010851	Myd88	Myeloid differentiation primary response gene 88
Mm.256765	NM_008689	Nfkb1	Nuclear factor of kappa light polypeptide gene enhancer in B-cells 1
Mm.286753	NM_019492	Rgs3	Regulator of G-protein signaling 3
Mm.289739	NM_178804	Slit2	Slit homolog 2 (Drosophila)
Mm.38049	NM_021297	Tlr4	Toll-like receptor 4
Mm.1293	NM_013693	Tnf	Tumor necrosis factor
Mm.1258	NM_011609	Tnfrsf1a	Tumor necrosis factor receptor superfamily, member 1a
Mm.483369	NM_019418	Tnfsf14	Tumor necrosis factor (ligand) superfamily, member 14
Mm.248352	NM_021406	Trem1	Triggering receptor expressed on myeloid cells 1
Mm.190	NM_008510	Xcl1	Chemokine (C motif) ligand 1
Mm.3317	NM_010368	Gusb	Glucuronidase, beta
Mm.299381	NM_013556	Hprt	Hypoxanthine guanine phosphoribosyl transferase
Mm.2180	NM_008302	Hsp90ab1	Heat shock protein 90 alpha (cytosolic), class B member 1
Mm.343110	NM_008084	Gapdh	Glyceraldehyde-3-phosphate dehydrogenase
Mm.328431	NM_007393	Actb	Actin, beta
